# Ferroelectric Domain Walls for Environmental Sensors

**DOI:** 10.1021/acsami.5c04875

**Published:** 2025-08-04

**Authors:** Leonie Richarz, Ida Cathrine Skogvoll, Egil Ytterli Tokle, Kasper Aas Hunnestad, Ursula Ludacka, Jiali He, Edith Bourret, Zewu Yan, Antonius T. J. van Helvoort, Jan Schultheiß, Sverre Magnus Selbach, Dennis Meier

**Affiliations:** † Department of Materials Science and Engineering, 8018 Norwegian University of Science and Technology (NTNU), NO-7491 Trondheim, Norway; ‡ Department of Electronic Systems, Norwegian University of Science and Technology (NTNU), NO-7491 Trondheim, Norway; § Department of Physics, Norwegian University of Science and Technology (NTNU), NO-7491 Trondheim, Norway; ∥ Materials Sciences Division, 1666 Lawrence Berkeley National Laboratory, Berkeley, California 94720, United States; ⊥ Department of Physics, 27258 ETH Zurich, 8093 Zurich, Switzerland; ¶ Faculty of Physics, University of Duisburg-Essen, 47057 Duisburg, Germany

**Keywords:** ferroelectric domain walls, environmental sensors, domain-wall-based nanoelectronics, scanning probe microscopy, oxygen defects

## Abstract

Domain walls in ferroelectric oxides provide fertile ground for the development of next-generation nanotechnology. Examples include domain-wall-based memory, memristors, and diodes, where the unusual electronic properties and the quasi-two-dimensional nature of the walls are leveraged to emulate the behavior of electronic components at ultrasmall length scales. Here, we demonstrate atmosphere-related reversible changes in the electronic conduction at neutral ferroelectric domain walls in Er­(Mn,Ti)­O_3_. By exposing the system to reducing and oxidizing conditions, we drive the domain walls from insulating to conducting and vice versa, translating the environmental changes into current signals. Density functional theory calculations show that the effect is predominately caused by charge carrier density modulations, which arise as oxygen interstitials accumulate at the domain walls. The work introduces an innovative concept for domain-wall-based environmental sensors, giving an additional dimension to the field of domain wall nanoelectronics and sensor technology in general.

## Graphical abstract

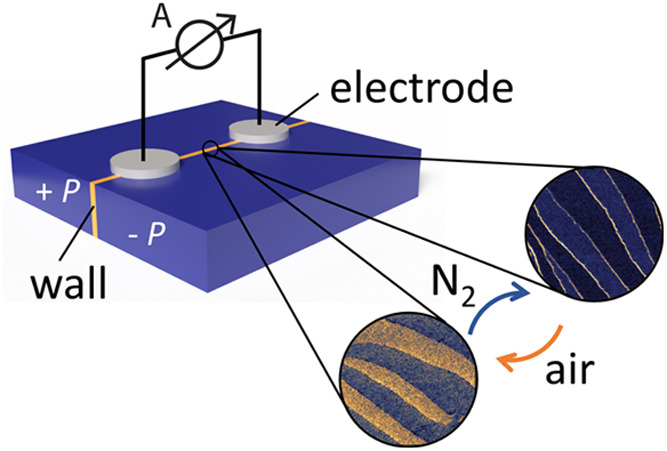

## Introduction

Ferroelectric domain walls attract broad attention as agile building blocks for nanoelectronics, offering unique functional properties and atomic-scale feature size.^1^ For example, it has been demonstrated that ferroelectric domain walls can be used to emulate the behavior of key electronic components and to control conductivity at the nanoscale,^2^ including digital switches,^3^ diodes,^4^ nonvolatile memory,^5^ and memristors.^6^ Furthermore, innovative, unconventional computing schemes have been proposed that utilize the responses of ferroelectric domain walls.^7^

In contrast to the extensive research on the design of such domain-wall-based electronic components, the interaction of domain walls with environmental conditions and related application opportunities in sensor technology have been much less explored. Environmental sensors play a key role in modern electronics and the Internet of Things, having profound implications for various fields ranging from home electronics and autonomous transport to safeguarding the ecosystem. The sensor’s task is to provide information about environmental factors, such as temperature, pressure, humidity, concentration of gases, and soil moisture, converting respective changes into electronic signals. One example is semiconductor-based gas sensors, which rely on conductivity variations that arise when a semiconducting oxide (e.g., SnO_2_ or ZnO) is exposed to a certain target gas.^8^ Other concepts include polymers, nanotubes, and two-dimensional (2D) materials for sensing applications.^9,10^

In this context, domain walls in ferroelectric oxides are particularly promising as they strongly interact with oxygen defects,^4,11–19^ which can be leveraged in sensing applications. Depending on the structure and charge state of a domain wall, it can either attract or repel oxygen defects and, thereby, amplify oxidation- and oxygen-reduction reactions.^4,11,20–22^ For example, it has been shown that oxygen vacancies have a tendency to accumulate at domain walls in BiFeO_3_ ^16,22,23^ and LiNbO_3_ ^24^ and codetermine their local electronic response. Vice versa, in BaTiO_3_, oxygen vacancies have been predicted to assemble in crystallographic planes, promoting the formation of charged domain walls.^19^ *In situ* microscopy studies indeed showed a strong correlation between the domain patterns and the annealing conditions that set the oxygen content,^17^ which provides a handle for tuning the density and positions of the domain wall.

In addition, domain walls can facilitate large ionic mobilities,^25^ further enhancing the reactivity and potentially reducing the recovery time after exposure to, e.g., oxygen-rich or -poor atmospheres. Most importantly for sensing applications, the direct relation between the concentration of oxygen defects at the domain walls and their electronic transport behavior allows one to readily translate environmentally driven variations in oxygen concentration into measurable changes in the local conductivity. As a first step toward a potential application in sensors, the enhanced conductance at ferroelectric domain walls in a LiNbO_3_ thin film was utilized to enhance the film’s response to changes in temperature.^26^ Similarly, the defect concentration at domain walls in LiNbO_3_ was shown to slow down electron–hole recombination, which is of interest for light sensing and in-memory computing.^27^

Here, we investigate the relation between reducing and oxidizing environmental conditions and the electronic transport properties at ferroelectric domain walls in Er­(Mn,Ti)­O_3_. We show that reversible changes in conductance occur at neutral domain walls when exposed to reducing and oxidizing atmospheres, changing the domain walls from insulating to conducting and vice versa. Our density functional theory (DFT) calculations reveal that oxygen-defect-driven variations in the local carrier concentration, rather than band gap-related effects, drive this change in transport behavior, providing a microscopic understanding. The results give additional insights into the interaction of oxygen defects and ferroelectric domain walls and demonstrate their general potential as active units for sensing applications, translating environmental parameters into measurable electronic signals.

## Exposure to Reducing Atmosphere

For our study, we use the ferroelectric semiconductor Er­(Mn,Ti)­O_3_ with 0.2% Ti doping (see Experimental Section for more information about the crystal). The moderate Ti doping is known to enhance oxygen kinetics,^28^ while maintaining the hexagonal crystal structure and domain wall's electronic properties.^29^ Also, for this material, the basic domain wall physics are well-understood,^11,30–38^ and it is predicted that the system has a propensity to accumulate oxygen interstitials at its neutral domain walls.^4,11^ In general, the system displays an outstanding chemical flexibility and tunable semiconducting transport properties (p-type^29^) that can be controlled via the oxygen content, i.e., the oxygen off-stoichiometry in Er­(Mn,Ti)­O_3+δ_, typically with δ > 0 in the as-grown state.^39–42^ This chemical flexibility, in combination with the affinity to accumulate oxygen interstitials at the neutral domain walls, makes it an ideal model system for domain-wall-based environmental sensing.

Er­(Mn,Ti)­O_3_ is one of the hexagonal manganites (*R*MnO_3_ with *R* = Sc, Y, In, Dy to Lu), in which the spontaneous polarization appears as a secondary effect, caused by a structural instability of the paraelectric high-temperature phase (*T*_C_ ≈ 1170 K^43^) that leads to a tripling of the unit cell and the formation of a polar axis^44^ (improper ferroelectricity^45,46^ with a spontaneous polarization *P* ≈ 5.6 μCcm^–2^).^45^ The structural instability drives the formation of topologically protected structural vortex lines that govern the domain formation and serve as anchor points for the ferroelectric domain walls, as explained elsewhere.^44^ As a consequence, a robust three-dimensional (3D) network of interconnected domain walls arises that we will explore in the following for the development of ultrasmall sensors.

We begin by analyzing the general impact of variations in oxygen off-stoichiometry on the domain wall conductance by exposing our sample to reducing conditions. Figure 1(a) shows a representative conductive atomic force microscopy (cAFM) scan recorded on the [001]-surface of an Er­(Mn,Ti)­O_3_ single crystal (*P* out-of-plane) before exposure. The cAFM map is recorded with a bias voltage, *V*_bias_, of 15 V applied to the back electrode while the probe tip is grounded (see Experimental Section for further details) and shows the characteristic transport behavior of [001]-oriented hexagonal manganites.^47^ Due to the screening of negative bound charges at the surface of −*P* domains by mobile hole carriers and barrier effects at the tip-sample interface, a higher conductance (bright) is measured on −*P* domains compared to the lower conductance (dark) on +*P* domains.^47^

**Figure 1:**
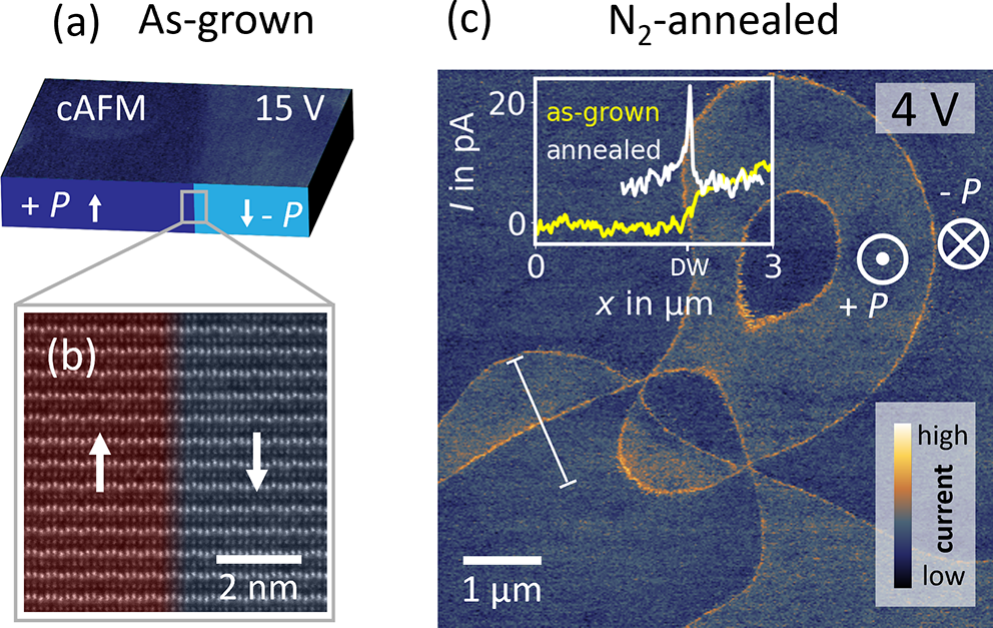
Domain wall conductance and annealing in a reducing atmosphere. (a) Illustration of the transport behavior of +*P* and −*P* domains in Er­(Mn,Ti)­O_3_ (out-of-plane polarization). The top corresponds to a cAFM map (*V*_bias_ = 15 V), showing lower (dark) and higher (bright) conductance in +*P* and −*P* domains, respectively. Polarization directions are indicated by white arrows. (b) Representative HAADF-STEM image of a neutral domain wall with color overlay (red: +*P*, blue: −*P*). (c) cAFM scan (*V*_bias_ = 4 V) obtained on the same sample as in panel (a) after annealing in N_2_ at 300 °C for 48 h. For consistency, the length and current scales are identical in panels (a) and (c); cAFM scans are performed with a diamond-coated DEP01 tip in ambient conditions at room temperature. Representative line plots comparing the conductance evolution between +*P* and −*P* domains in panels (a) and (c) are shown in the inset of panel (c).

The +*P* and −*P* domains in Figure 1(a) are separated by a neutral 180° domain wall as highlighted by the representative high-angle annular dark-field scanning transmission electron microscopy (HAADF-STEM) image in Figure 1(b). The HAADF-STEM image is recorded viewing along the [110̅] direction, with bright dots indicating the positions of the Er atoms and shows the characteristic atomic-scale structure of a neutral domain wall in Er­(Mn,Ti)­O_3_.^38^ The local polarization direction can readily be determined based on the Er displacement patterns (+*P* = "up-up-down" and −*P* = "down-down-up") as explained in refs 38,48 . Most importantly for this work, the cAFM data in Figure 1(a) show that in the as-grown state, the transport properties at the neutral domain wall are similar to the bulk and cannot be distinguished from the surrounding domains.

Figure 1(c) presents a conductance map gained on the same sample with a 4 V bias voltage after exposing the sample to reducing conditions. More specifically, the post-annealing cAFM image is taken in ambient conditions at room temperature after transferring the sample from the annealing furnace to the AFM. In the furnace, the sample was annealed in nitrogen (N_2_) at 300 °C for 48 h (heating/cooling rate: ±200 °C/h). The transfer from the furnace to the AFM took about 30 min, during which the sample was exposed to ambient conditions. The most pronounced effect is a qualitative change regarding the transport behavior at the neutral domain walls. We note that cAFM is a two-point technique, which probes a convolution of intrinsic and extrinsic conduction effects.^49^ Thus, we focus on relative changes observed in cAFM conductance maps, comparing local domain and domain wall currents. The measured differences in conductance represent a qualitative measure that can be utilized for sensing, making the domain-wall-based sensor robust against global variations in bulk conductivity. After exposure to reducing conditions, the conductance at the domain walls is about three times higher than in the domains, reflecting that the neutral domain walls have a much higher sensitivity to the environmental history than the domains. A control experiment using Ar annealing yields qualitatively similar changes in domain wall conductance (see Figure S1). This observation corroborates the conclusion that the behavior is governed by the gradient in oxygen partial pressure and not specific to nitrogen atmosphere.

## Reversibility of Environmentally Driven Effects

In the next step, we explore to what extent the domain wall conductance can be reset to the initial state and test the repeatability of the process. For this purpose, we introduce an additional heating step at up to 200 °C involving synthetic air (labeled “air”) as summarized in Figure 2 (see Supporting Table S1 and Experimental Section for details).

**Figure 2:**
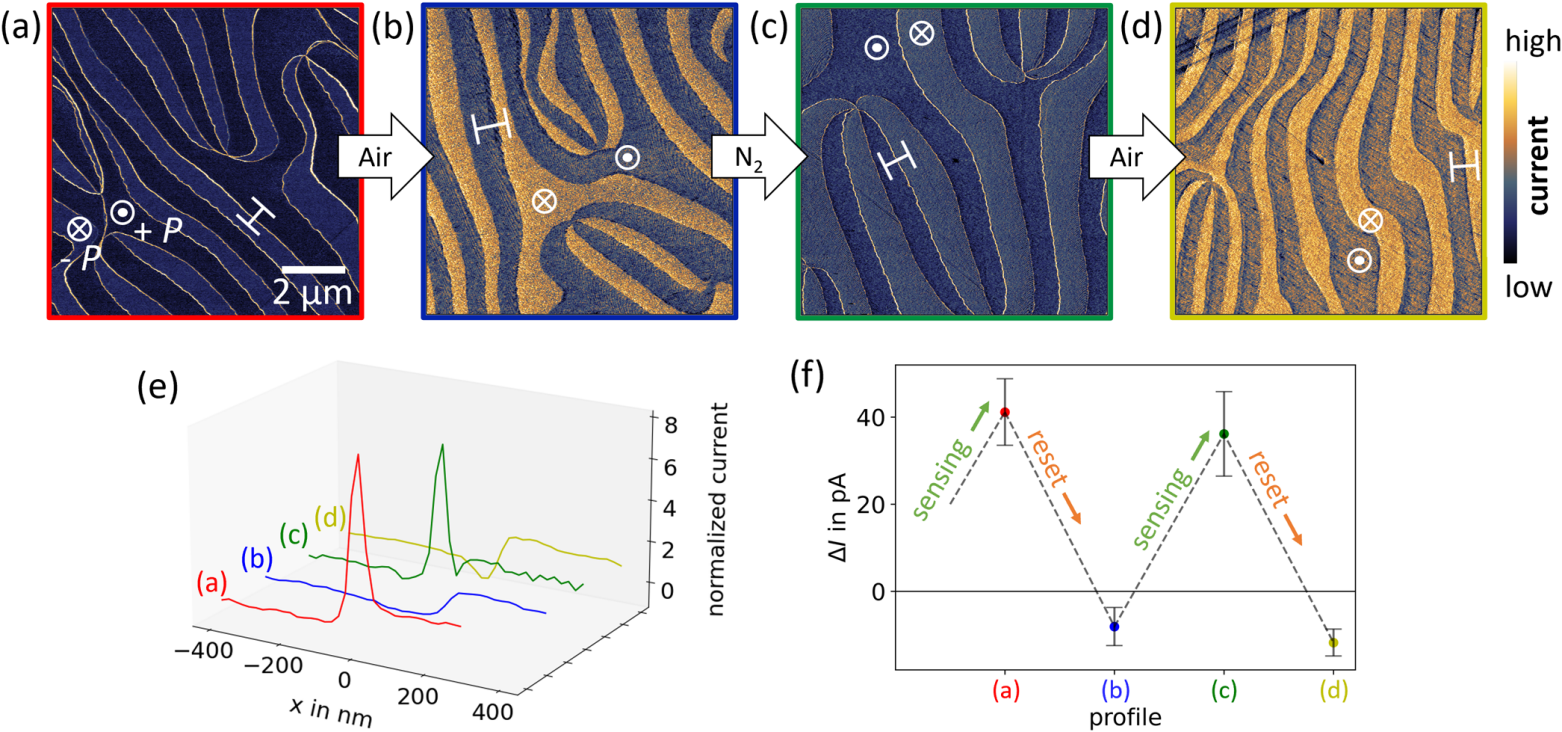
Reversibility of atmosphere-driven changes in domain wall conductance. cAFM scans taken (a) after annealing in N_2_ at 300 °C for 48 h, (b) after subsequent heating up to 200 °C in N_2_ and synthetic air, (c) after repeated annealing in N_2_ at 300 °C for 48 h, and (d) after repeated heating up to 200 °C in N_2_ and synthetic air (see Supporting Information for details). After annealing, the samples were exposed to ambient conditions for ≤ 30 min before the cAFM scans started. (e) Current profiles extracted along the white markings in (a–d). The profiles are normalized such that the +*P* (−*P*) domains have an average current value of 0 (1). (f) Relative domain wall conductance Δ*I* for the four profiles in panel (e). By exposing the system to different atmospheres, Δ*I* switches between conductive (Δ*I* ≫ 0) and insulating (Δ*I* < 0) behavior. The cAFM scans are performed with diamond-coated tips DEP01 for panels (a–c) and CDT-NCHR for panel (d) and bias voltages of (a) 2 V, (b) 4 V, (c) 5 V, and (d) 5 V. All cAFM scans are performed at room temperature in air. Data taken in different regions of the sample show qualitatively equivalent results regarding the annealing-driven changes in conductance (Figure S2), confirming the effect across the entire sample surface.

The cAFM data in Figure 2 show conductance maps that were taken on the same sample while going through consecutive annealing cycles. To avoid potential imprints from previous scans, the cAFM maps are recorded in different positions as seen from the different domain structures in Figure 2(a–d). Figure 2(a) shows a representative conductance map (*V*_bias_ = 2 V) obtained after annealing in a reducing atmosphere (N_2_) at 300 °C, following the same annealing procedure as in Figure 1. Bright features in the cAFM image correspond to neutral domain walls, showing that their conductance is substantially higher than in the +*P* and −*P* domains after exposure to the reducing environmental conditions.

The conductance map in Figure 2(b) presents the transport properties after an additional annealing step involving synthetic air. We find that the sample exhibits a pronounced contrast between +*P* and −*P* domains (*V*_bias_ = 4 V), whereas no specific cAFM signal is measured from the domain walls, which is qualitatively the same behavior as that in the as-grown state shown in Figure 1(a). This observation leads us to the conclusion that the changes in domain wall conductance induced by the exposure to reducing atmospheres are reversible and that the initial conditions can be restored by applying adequate annealing procedures. As shown by Figure 2(a–d), this process is repeatable, leading to an activation and deactivation of the enhanced transport behavior at the neutral domain walls, which is a key characteristic for the development of domain-wall-based sensors.

The same changes in domain wall conductance can also be achieved by alternating annealing in nitrogen, which triggers the onset of conductance, and pure oxygen, which inhibits the conductance. An example is presented in Figures S3 and S4, which qualitatively exhibits the same behavior as seen in Figures 1 and 2. These measurements support the conclusion that the behavior is universal, i.e., not specific to a particular sample or surface area. The cAFM scans lead us to the conclusion that changes in oxygen off-stoichiometry are the driving mechanism for the observed changes in the domain wall conductance.

To quantify the changes in domain wall conductance, we analyze local current profiles extracted from the data in Figure 2(a–d). The profiles are displayed in Figure 2(e) and show that the current at the neutral domain walls in Figure 2(a,c) is about five to six times higher than for the −*P* domains, whereas a slightly reduced current signal is observed for the walls in Figure 2(b,d). Figure 2(f) displays this change in relative domain wall conductance, Δ*I*, for the consecutive annealing steps, going back and forth between conductive (Δ*I* > 0) and insulating (Δ*I* < 0) domain walls (see Experimental Section for details on the data analysis). The data thus establish a one-to-one correlation between the atmospheric conditions to which the sample was exposed and the domain wall conductance, translating environmental changes into measurable conductance changes. Based on the kinetic effects discussed in ref. 28, we expect the domain walls to readily react to variations in atmosphere, changing their conductivity on the time scale of seconds. Equilibrium is reached again after approximately 1 min at 300 °C and 15 min at 200 °C, primarily governed by surface oxygen exchange. Here, we define the onset of enhanced domain wall conductance as the “sensing” step and its suppression as “reset”, inspired by the functional behavior required for the realization of a domain-wall-based oxygen sensor, which is triggered (switches from insulating to conducting) when the environmental oxygen level falls below a certain threshold value, as we emulated by the exposure to reducing conditions.

## Microscopic Origin

DFT calculations presented in previous works have shown that the emergence of p-type conductance at neutral domain walls in hexagonal manganites correlates with the local density of oxygen interstitials.^4,11,21,42^ Within this picture, there are two basic effects that can explain the observed annealing-induced enhancement of the domain wall conductance (see Figure 2), i.e., (i) a more pronounced loss of oxygen interstitials in +*P* and −*P* domains compared to the domain walls during N_2_-annealing or (ii) a more efficient reoxidation at the domain wall after annealing in N_2_. Consistent with the reduced defect formation energy for oxygen interstitials at the neutral domain walls, both effects lead to an increase in oxygen concentration relative to the domains and, hence, can contribute to the observed enhancement of the local conduction.^4,11,21^

The question that remains to be answered is how such changes in the oxygen concentration impact the electronic structure, clarifying the microscopic mechanism for enhanced domain wall conductance. To gain insight into the driving mechanism for the enhanced conductance at the neutral domain walls, we perform DFT calculations for different concentrations of oxygen interstitials at domain walls in ErMnO_3_ and determine the density of states (DOS) as presented in Figure 3. ErMnO_3_ consists of alternating Er and Mn-O layers with corner-sharing trigonal bipyramids of Mn^3+^ and O^2–^. The energetically most favorable position for oxygen interstitials is at one of the six equivalent lattice sites between the Mn atoms in the Mn-O planes^42^ (see Supporting Information for details of the DFT calculations).

**Figure 3:**
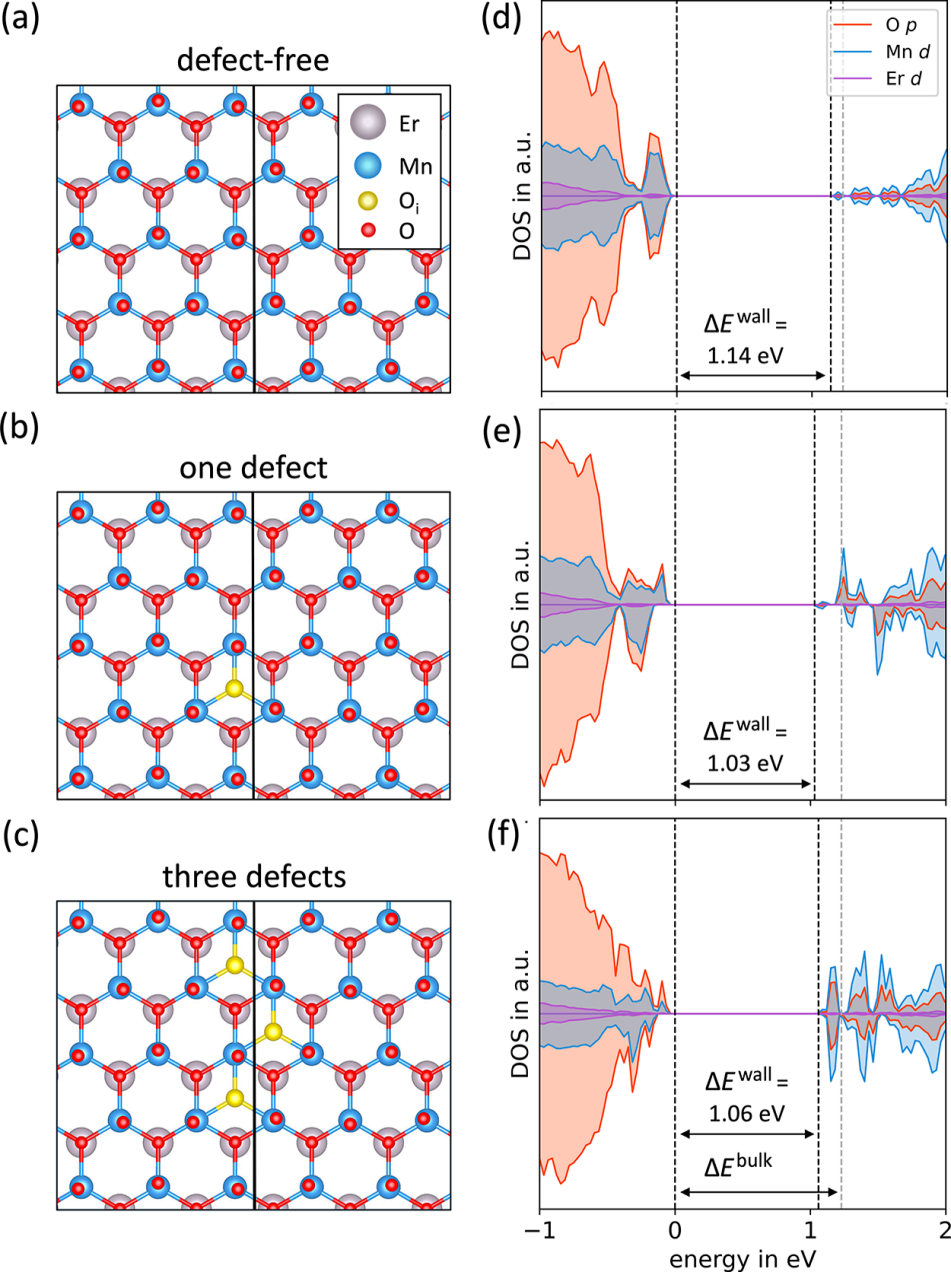
Relation between defect density and electronic structure at domain walls. (a–c) Schematics of the crystal structure of ErMnO_3_ with (a) none, (b) one, and (c) three oxygen interstitials at the domain wall within our simulation cell. (d–f) The density of states is calculated for the scenarios illustrated in panels (a–c). Dashed lines represent the Fermi level and the bottom of the conduction band in pristine ErMnO_3__'_ and Δ*E* ^wall^ denotes the band gap. The defects illustrated in panel (c) are distributed over two layers for the calculations.

Figures 3(a,d) show the model of a neutral domain wall and the calculated DOS. Consistent with literature,^4,21,42^ we observe qualitatively the same electronic structure as in the bulk with only a subtly smaller band gap (i.e., Δ*E* ^bulk^ = 1.23 eV and Δ*E* ^wall^ = 1.14 eV). When introducing one oxygen interstitial at the domain wall, corresponding to a local off-stoichiometry of δ = 0.08 (where δ is defined in terms of the volume encompassed by the domain-wall width), a localized and nonbonding defect state arises at the bottom of the conduction band, with the corresponding bonding states situated at the bottom of the valence band (Figure 3(b,e)). This effect is caused by a small shift of the oxygen interstitial distance away from its central position, changing the valence state of two Mn atoms from Mn^3+^ to Mn^4+^. This implies that the electronic transport at the neutral domain walls occurs through a p-type polaron hopping mechanism.^42^ We also observe a lowering of the band gap to Δ*E* ^wall^ = 1.03 eV, which is 0.11 eV lower than that for a defect-free domain wall, while a single defect in bulk leads to a considerably smaller band gap of 0.81 eV. This can explain the observed disparity in the relative bulk-wall conductance after annealing in a reducing atmosphere versus synthetic air, as the bulk has an additional effective charge carrier contribution from the band gap reduction, even at lower defect concentrations. As the concentration of oxygen interstitials at the neutral domain wall increases to δ = 0.24 in Figure 3(c,f), the defect states become less localized, and the band gap remains close to the value for one defect.

A polaron hopping mechanism is inferred at the domain wall from the DFT results. The charge carrier density modulation, caused by hole charge-compensating oxygen interstitials, is identified as the main reason for the enhanced domain wall conductance in the scans shown in Figures 1 and 2. While the presence of interstitials at domain walls also results in a smaller local band gap and minor changes to the electron/hole mobility, these effects are too small to account for the experimental observations in Figure 2.

## Outlook

The results presented in Figures 1 to 3 show a direct relation between environmental conditions (here, the concentration of oxygen in the atmosphere) and the electronic conduction at neutral ferroelectric domain walls in Er­(Mn,Ti)­O_3_. All experiments were conducted on domain wall networks at the surface of millimeter-sized single crystals. While it is clear that the physical and chemical domain wall properties will not change qualitatively upon down-scaling,^50^ it remains to be demonstrated that smaller volumes can be prepared while preserving the domain walls. To test the general feasibility, we use scanning electron microscopy (SEM) in combination with a focused ion beam (FIB) to extract a volume of 2 × 2 × 8 μm^3^ with an individual domain wall as displayed in Figure 4(a). The SEM image shows the isolated specimen with one domain wall in the center, separating two ferroelectric domains (bright and dark) of opposite polarization.

**Figure 4:**
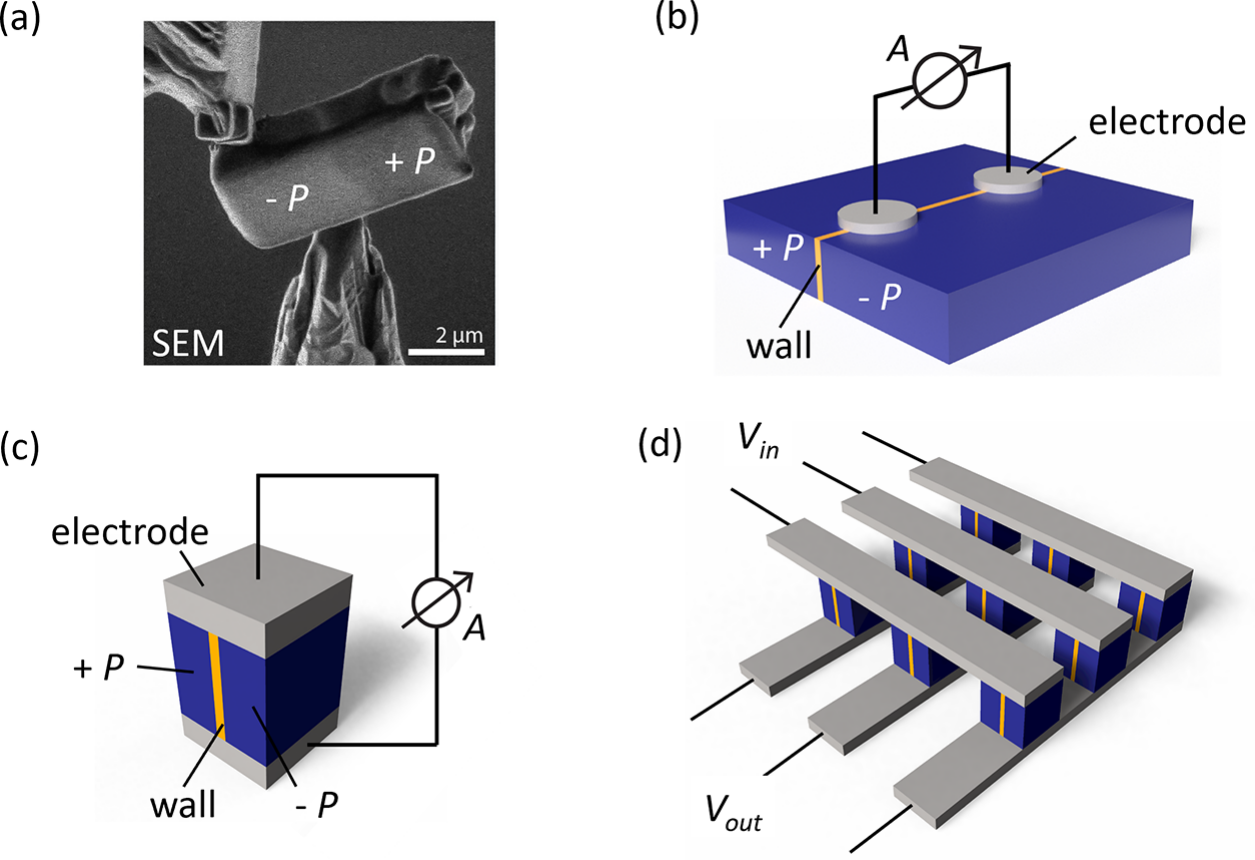
Concept for domain-wall-based sensors. (a) SEM image of a volume with a single ferroelectric domain wall extracted from a bulk sample using FIB, demonstrating the general possibility to isolate individual walls for sensor development. (b, c) Illustration of different sensing geometries based on a single ferroelectric domain wall using lateral (b) or transversal (c) electrode arrangements. (d) Crossbar geometry combining multiple transversal electrode arrangements into an array for spatially resolved sensing.

On the one hand, the successful extraction of a single domain wall demonstrates the possibility to scale domain-wall-based sensors and work with individual walls. On the other hand, it shows the opportunity to apply different geometries as illustrated in Figure 4(b,c). For example, electrodes can be patterned on the sample surface to measure environmentally driven changes in domain wall resistivity, as outlined in Figure 4(b). Alternatively, micrometer-sized sensor units may be realized, as sketched in Figure 4(c), which may be used individually or in crossbar arrangements (Figure 4(d)), enabling spatially resolved sensing. Independent of the specific geometry, the domain walls represent the active sensing medium, switching from insulating to conducting (or vice versa) as a function of the environmental conditions, as demonstrated in Figure 2.

## Conclusions

In conclusion, the results presented in this work demonstrate the reversible control of the electronic transport properties at neutral ferroelectric domain walls in Er­(Mn,Ti)­O_3_. By exposing the walls to reducing and oxidizing atmospheres, their conductance can be reversibly switched between insulating and conducting behavior, translating changes in atmospheric conditions into electronic signals. The effect originates from the distinct interaction of oxygen defects with the domain walls, which can readily be expanded toward other oxide systems.^16,22,24^

We note that despite extensive attempts using state-of-the-art chemical analysis techniques, such as atom probe tomography, time-of-flight secondary ion mass spectroscopy, and electron energy loss spectroscopy, we did not resolve statistically significant anomalies in the oxygen concentration at the domain walls (not shown). However, as these measurements are conducted in vacuum, that is, under highly reducing conditions, it is reasonable to assume that the experiments themselves altered the concentration of oxygen defects and, hence, cannot be used to gain quantitative insight. The latter is a general experimental challenge when studying local oxygen defect levels in ferroelectric oxides^51^ and additional possibilities may arise from the ongoing progress in cryogenic microscopy, allowing to suppress vacuum- and beam-related oxygen loss.^52^

Our proof-of-concept experiments demonstrated the general possibility of cycling between insulating and conducting domain wall states (up to four annealing cycles), which is essential for going beyond single-use sensors. In the next step toward applications, it will be important to also investigate the long-term performance over larger numbers of annealing cycles, exploring the opportunities and limitations associated with domain-wall-based sensors. In this work, we selected moderately Ti-doped Er­(Mn,Ti)­O_3_ with enhanced oxygen kinetics compared to ErMnO_3_ for establishing the concept of domain-wall-based sensing. The compositional flexibility of the system allows for a wide range of doping levels, giving additional opportunities for tailoring the performance. For example, based on the established defect chemistry of the hexagonal manganites,^53^ we anticipate that more resilient (lower doping level) or receptive (higher doping level) oxygen sensors can be achieved. This possibility reflects that we have only scratched the surface with our proof-of-concept experiments, foreshadowing yet-to-be-explored opportunities and a large playground for the development of domain-wall-based sensors. Our approach is universal in the sense that it operates in any reducing and oxidizing atmosphere, with the sensitivity determined by the specific current detection scheme employed. The findings provide a basis for the design of domain-wall-based environmental sensors and give an additional dimension to the field of domain wall nanoelectronics, expanding the pool of functional materials for sensor technology toward ferroelectric domain walls.

## Experimental Section

### Synthesis and Sample Preparation

Er­(Mn,Ti)­O_3_ single crystals are grown by the pressurized floating zone method.^54^ The samples are then oriented by Laue diffraction and cut such that the *c*-axis is perpendicular to the sample surface ([001]-oriented), resulting in an out-of-plane polarization where the domain polarization can be either pointing out of the sample surface (+*P*, up) or into the sample surface (−*P*, down). The crystals are lapped with a 9 μm-grained Al_2_O_3_ water suspension and polished with a silica slurry, resulting in a surface roughness of approximately 0.5 nm.

### Microscopy

The cAFM scans are collected using a commercial AFM (Asylum Research, Cypher ES Environmental AFM). Diamond-coated tips are used, and the cAFM maps are conducted with the positive bias voltage, *V*_bias_, applied to the back electrode while the tip is grounded. FIB cutting and SEM imaging were performed using a Thermo Fisher Scientific G4UX Dual-beam FIB-SEM.

### Annealing and Heating

The annealing is performed in an Entech Tube furnace with a cooling/heating rate of 200 °C/h and a maximum temperature of 300 °C. The dwell time at the maximum temperature is 48 h. Before the annealing, the furnace is evacuated three times below 0.1 mbar to ensure a pure annealing atmosphere. A continuous gas flow is maintained during all steps of the annealing. The gas flow was visually monitored by using a flow meter (Swagelok, Solon, OH) with a flow rate of 0.2–0.4 NL/min under atmospheric pressure.

Additional heating experiments are performed inside the Cypher ES Environmental AFM. The sample chamber is first flushed with the desired gas, and then an overpressure of ≈300 mbar is built up to avoid contaminants from the outside. Annealing experiments in the AFM are performed at temperatures up to 250 °C, which is the upper limit in our setup. Detailed heating procedures can be found in the Supporting Information.

### Data Analysis

The relative domain wall conductance shown in Figure 2(f) is obtained from the profiles in Figure 2(e). The profiles are averaged over a width of 290 μm to reduce the influence of noise in the scan data. To make the profiles comparable, the current values are normalized such that the darker domain has an average current of 0 and the brighter domain of 1. For this, all current values are transformed as $I^{\prime }=\frac{I-I_{\mathrm{dark}}}{I_{\mathrm{bright}}-I_{\mathrm{dark}}}$ , For deriving the normalized domain wall current, the curves are fitted with a function of the form

1 $$I\left(x\right)=\underset{I_{\mathrm{step}}\;\left(x\right)}{\underset{︸}{\frac{A_{s}\;}{1+e^{-2\cdot \sigma_{s}\;\cdot \left(x-x_{0}^{s}\;\right)}\;}}}+\underset{I_{\mathrm{peak}}\;\left(x\right)}{\underset{︸}{A_{g}\;\cdot e^{-{\left(x-x_{0}^{g}\;\right)}^{2}\;/\sigma_{g}^{2}\;}\;}}+C$$

where *I*_step_ represents the difference in conductance between the two domain states, whereas *I*_peak_ represents the enhanced or reduced conductance at the domain wall. The distance along the profile line is represented as *x*. From this fit, the height of the additional current change at the domain wall, Δ*I* = *A*_g_, can be derived for all four profiles.

### Density Functional Theory

DFT calculations were done using the VASP^55,56^ code with the PBEsol functional^57^ and GGA + *U*,^58^ to which a Hubbard *U* of 4 eV was applied to the Mn 3*d* orbitals. The PAW method^59^ with a cutoff energy of 550 eV was used together with the Er_3, Mn_pv, and O pseudopotentials supplied with VASP. A Γ-centered 4 × 4 × 2 *k*-point mesh was used for the 30-atom unit cell, with similar densities for larger supercells. For bulk calculations, atomic positions were relaxed until the residual forces were below 0.001 eV/Å for the 30-atom cell and 0.005 eV/Å for the 2 × 2 × 1 supercell. For neutral domain walls, a 1 × 6 × 1 supercell was used for the pristine wall and a 2 × 6 × 1 supercell was used with defects present. The geometry was optimized until the forces on the atoms were below 0.02 eV/Å. The noncollinear magnetic order on the Mn atoms was approximated by a frustrated collinear antiferromagnetic order^60^ and kept continuous across the domain walls (see Supporting Information for details on magnetic order in the defect calculations). Band structure unfolding was done using the easyunfold package.^61^ Details on DOS and band structure calculations are provided in Supporting Figures S5 to S11.

## Supplementary Material



## Notes

Conductance data after annealing in pure atmosphere, annealing history, DOS and band structure calculations (PDF)
