# Supplementary material for: Ferroelectric Domain Walls for Environmental Sensors
Source: ACS Appl Mater Interfaces. 2025 Aug 4;17(33):47576–84. doi: 10.1021/acsami.5c04875 (PMC12371688; doi:10.1021/acsami.5c04875)
Supplement: Supplementary file 1 [file am5c04875_si_001.pdf]

Supporting Information: Ferroelectric domain walls for environmental sensors

Leonie Richarz,[†] Ida Cathrine Skogvoll,[†] Egil Ytterli Tokle,[†] Kasper Aas Hunnestad,[†,§] Ursula Ludacka,[†] Jiali He,[†] Edith Bourret,[¶] Zewu Yan,[¶,‖] Antonius T.J. van Helvoort,[‡] Jan Schultheiß,[†] Sverre Magnus Selbach,[†] and Dennis Meier[*,†]

† *Department of Materials Science and Engineering, Norwegian University of Science and Technology (NTNU), NO-7491 Trondheim, Norway*

‡*Department of Physics, Norwegian University of Science and Technology (NTNU), NO-7491 Trondheim, Norway*

¶*Materials Sciences Division, Lawrence Berkeley National Laboratory, Berkeley, CA 94720, USA*

§*Department of Electronic Systems, Norwegian University of Science and Technology (NTNU), NO-7491 Trondheim, Norway*

‖*Department of Physics, ETH Zurich, 8093 Zurich, Switzerland*

E-mail: dennis.meier@ntnu.no

Annealing in different atmospheres

The onset of domain wall conductance after annealing, demonstrated in Figure 1, can also be achieved by annealing in other reducing atmospheres such as Ar, as can be seen in Figure S1(c). Annealing in oxidizing atmospheres, on the other hand, does not result in conductive domain walls (see Figure S1(d)).

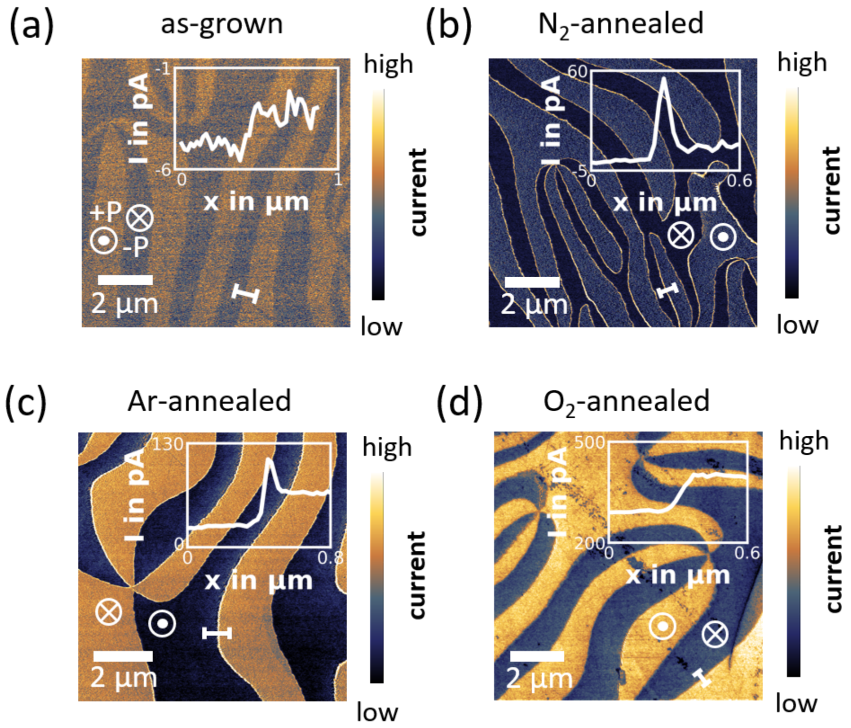

Figure S1: Comparison of the conduction properties of the as-grown Er(Mn,Ti)O_3 sample in (a), with samples annealed in (b) nitrogen, (c) argon and (d) oxygen. The inserts show the current profiles along the white line for the respective scan. All samples are annealed for 48 h at 300 °C with 200 °C/h heating/cooling rate. The scans are collected with diamond coated DEP01 tips at (a) 20 V, (b) 3 V, (c) 4 V and (d) 4 V.

Annealing History

As discussed in the main text, the reversible switching of the conductance at neutral domain walls is a non-trivial process, as the gradient of the oxygen off-stoichiometry has to be precisely controlled to avoid large-scale switching of the surface domains.

The furnace annealing in N_2 is always performed at 300°C, while the temperature range of the heating inside the AFM is limited to 250°C. Previous studies have shown that O_2 annealing above 200°C can result in oxygen loss in the hexagonal manganites.[1] The limited temperature range of the AFM heating assures that we stay below this anomaly in oxygen off-stoichiometry.

Table S1 describes the full heating history of the sample used for collecting the scans shown in Figure 2. The scans in Figure 2 are taken before (a) and after (b) the heating on day 9, as well as before the heating on day 45 (c) and after the heating on day 50 (d).

Table S1: Annealing history of the sample shown in Figure 2. T_{max} describes the maximum temperature that was reached during the heating, the time gives the approximate time the sample spent at that maximum temperature, and the rate gives the cooling and heating rate. Many of the heating experiments in the Cypher AFM were performed stepwise, and no absolute heating rate can be given. The last column describes the relative domain wall conductance after the respective heating cycle, determined from cAFM measurements carried out directly after the respective annealing of the sample.

Day	Atmosphere	T_{max} in °C	Time in h	Rate in °C/h	Instrument	Domain walls
1	N_2	300	48	200	Entech Tube Furnace	conducting
3	N_2	200	2	1800	Cypher AFM	conducting
9	N_2 & Air	200	2	stepwise	Cypher AFM	not conducting
15	N_2	250	2	1800	Cypher AFM	not conducting
28	N_2	300	48	200	Entech Tube Furnace	conducting
43	N_2	100	1	stepwise	Cypher AFM	conducting
45	N_2	200	0.5	stepwise	Cypher AFM	conducting
50	N_2 & Air	200	1	stepwise	Cypher AFM	not conducting

For each data point, scans were collected at multiple scan areas, distributed over the whole sample surface. A representative selection of such scan points at the annealing steps shown in Figure 2, are presented in Figure S2.

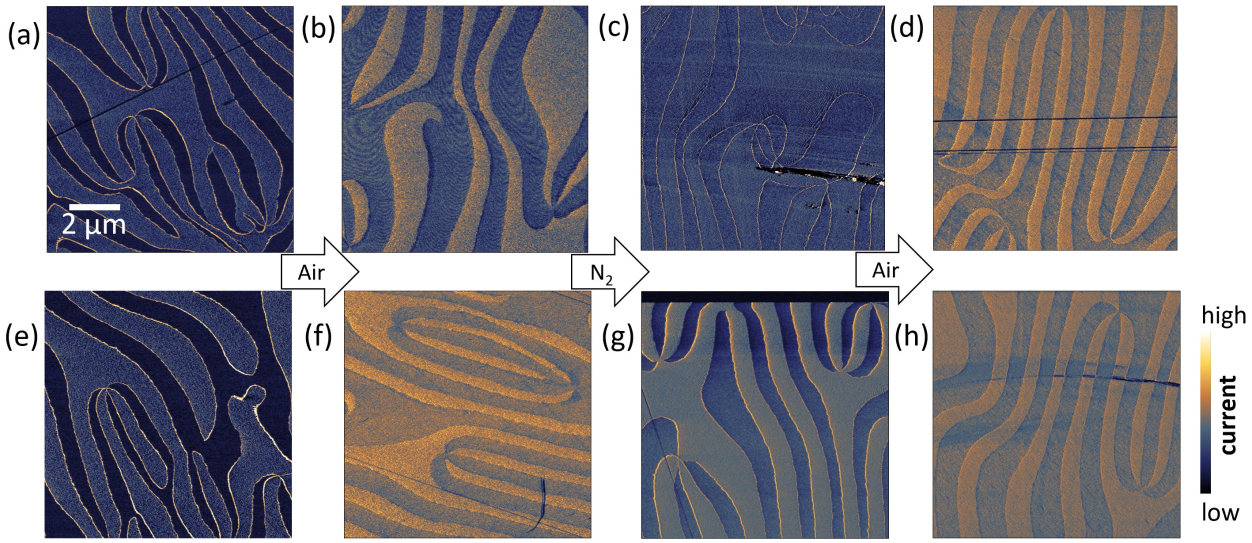

Figure S2: Scans collected at the different annealing steps presented in Figure 2. The scans are collected at different areas distributed over the whole sample surface. The cAFM scans are collected using a diamond-coated DEP01 tip (all scans) with 3 V (a,d,e), 5 V (c,g,h) and 4 V (b,f) applied to the back-electrode.

Reversibility in N_2-O_2-N_2-Annealing

To see if a transition from conducting to insulating domain walls (shown in Figure 2 in the main text) can also be achieved by pure oxygen annealing, we perform additional annealing experiments. An N_2-annealed sample is further annealed in O_2 at 300 °C for 48 hrs with 200 °C/h cooling and heating rate. Profiles across a domain wall after annealing in N_2 and after the subsequent O_2 annealing are shown in Figure S3(a) and (b) respectively. As visible, the domain wall conductance is clearly enhanced after N_2 annealing. After the following O_2 annealing, no enhanced domain wall conductance is visible any more. This shows that the transition from conducting to insulating domain walls by in-situ heating that was shown in Figure 2 in the main text can also be achieved by annealing in O_2. Subsequent annealing in N_2 restores the enhanced conductance at the domain walls (see Figure S3), again demonstrating the reversibility of the process.

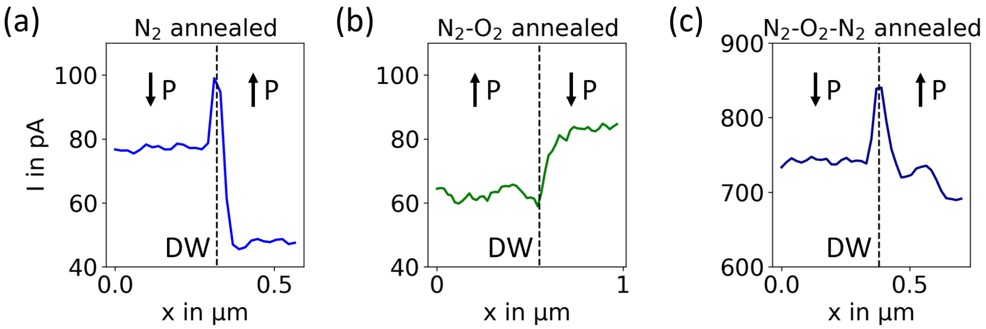

Figure S3: Profiles across a domain wall (approximate position marked by the black dashed line) after different steps of the annealing cycle. (a) After the sample was annealed in N_2 a clear increase in conductance is visible at the domain wall. (b) After subsequent O_2 annealing no enhanced conductance at the wall can be observed. (c) After the sample is annealed in N_2 again, the domain wall shows increased conductance. The corresponding cAFM scans can be found in Figure S4.

This annealing cycle (N_2-O_2-N_2) is, however, accompanied by significant switching of the surface domains, as visible when comparing the current data in the corresponding cAFM scans presented in Figure S4(b) and (c) with the respective topography in Figure S4(e) and (f). The topography signal carries the information about the domain state in the as-grown

state due to selective etching during the sample preparation[2] and can thus be used as a reference for changes in the domain configuration. The switching behavior can be explained by a gradient in oxygen off-stoichiometry, where the annealing affects only the surface-near region.[3]

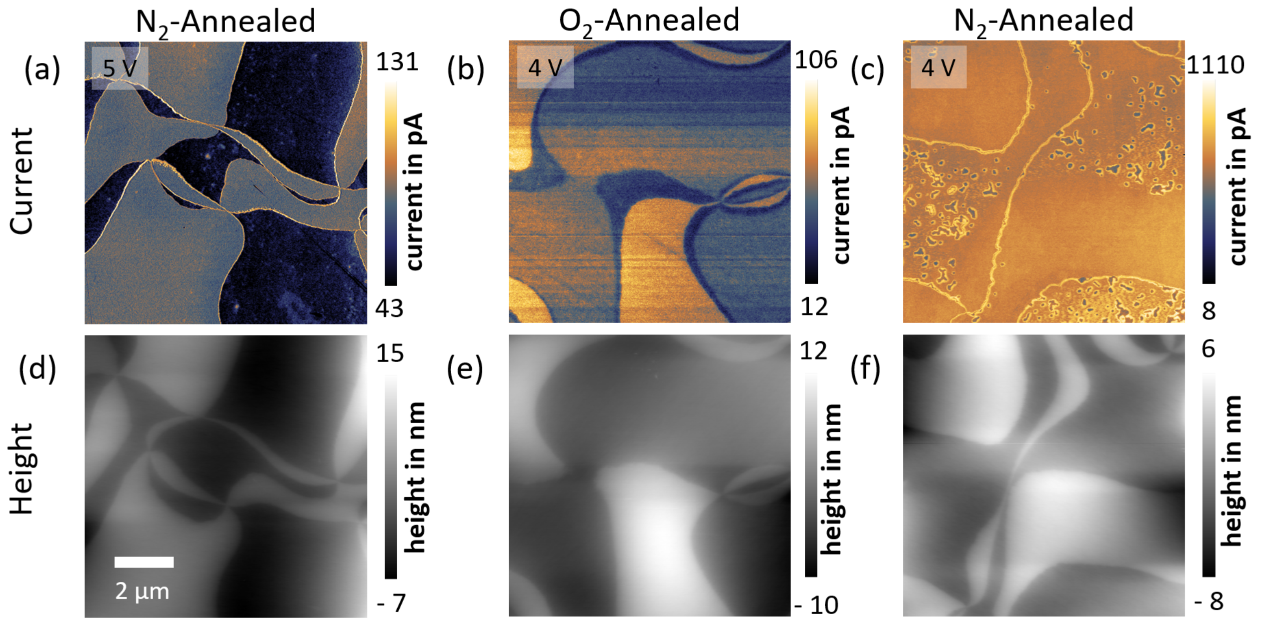

Figure S4: Comparison of the domains visible in the current (a)-(c) and the height channel (d)-(f) at different points in the annealing cycle. (a) and (d) are recorded after the sample is annealed in N_2 for the first time. (b) and (e) are recorded after the same sample is subsequently annealed in O_2. Data (c) and (f) are recorded after a subsequent second annealing in N_2. All images are recorded with a grounded, diamond-coated DEP01 tip and a voltage of (a) 5 V, (b) and (c) 4 V applied to the back electrode. Current and height are always recorded simultaneously but the positions on the sample are different for the different annealing steps to avoid scan imprints.

The initial N_2 annealing results in a slight expansion of the bright $-P$ domains (see Figure S4(a)). This effect is consistent with a downwards pointing electric field, induced by a reduction in the amount of negatively charged interstitials or introduction of positively charged vacancies at the surface. This is the expected case after annealing in reducing atmospheres. This slight variation in the domain size can be observed on most N_2 annealed samples, and the flexibility in the domain wall position would need to be taken into account for a potential sensor device. The subsequent O_2 annealing then leads to an expansion of

the darker $+P$ domains. Again, this is consistent with the expected increase in the amount of negatively charged oxygen interstitials at the surface, introducing an upwards pointing electric field close to the sample surface. The final N_2 annealing results in pronounced switching, where the dark $+P$ domains are contracted to thin lines and bubbles. Such a strong amount of switching has not been previously observed in the samples that were re-oxidized by in-situ heating (see Figure 2 in the main text for reference), indicating that oxidation and reduction during annealing of the samples have different dynamics. This highlights the importance of carefully controlling the annealing parameters if full reversibility is desired.

Density functional theory

The structure of $ErMnO_3$ consists of alternating Er and Mn-O layers in the *ab*-plane, with the latter forming corner-sharing trigonal bipyramids of Mn^{3+} and O. Within each layer, the two Er atoms in the Er2 position are displaced down while the single Er1 is displaced upwards in the *c*-direction, giving rise to the polarization, see Figure S5. The largest dispersion in the band structure of $ErMnO_3$ is observed in high-symmetry directions corresponding to intra-layer directions within the unit cell. The calculated band gap is $E_g = 1.23\,\mathrm{eV}$.

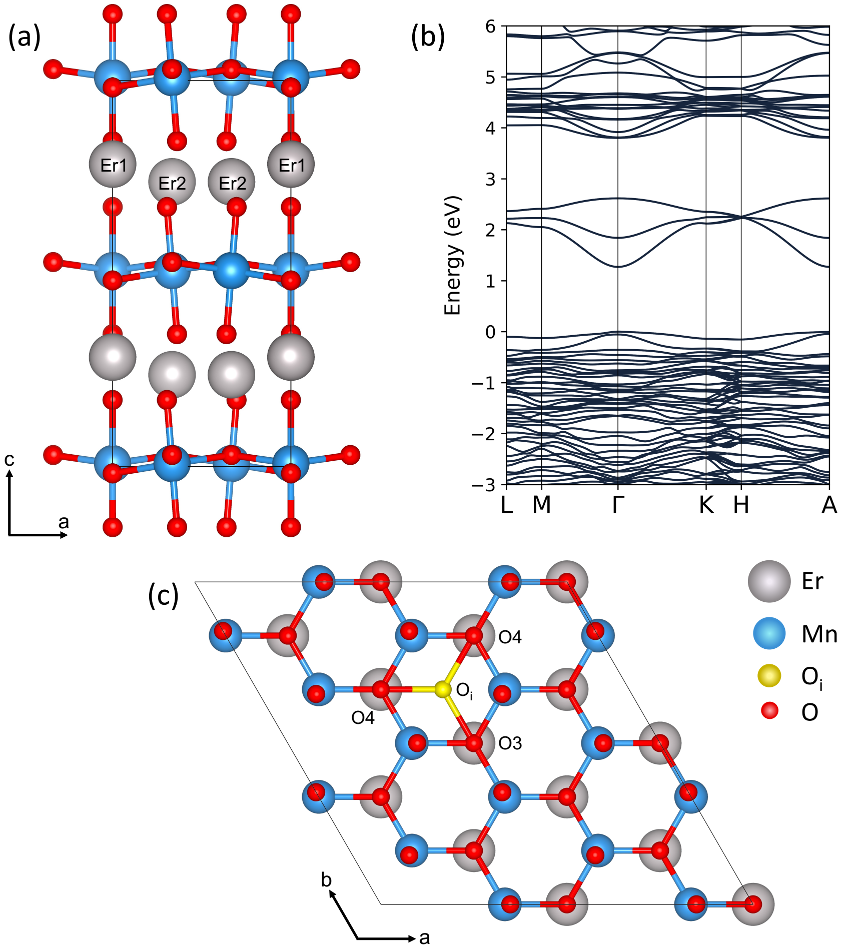

Figure S5: (a) The unit cell structure of $ErMnO_3$, with Mn atoms indicated in blue, O in red and Er in grey. (b) The band structure of $ErMnO_3$. The calculated band gap is $E_g = 1.23\,\mathrm{eV}$. (c) (001) plane of the unrelaxed $2 \times 2 \times 1$ supercell, with O_i in a stable position (yellow).

The most energetically stable position for interstitial oxygen (O_i) in the lattice is within

the Mn−O layers, centered between the three Mn atoms, such that there is six equivalent lattice sites within one unit cell, as shown in Figure S5(c). In this position, O_i experiences a triple well potential where each minima is shifted slightly from the central position towards two Mn atoms. These are then oxidized from Mn^{3+} to Mn^{4+} through a partial charge transfer, resulting in two Mn−O bond lengths of equal size and one elongated bond to the latter Mn atom. As shown in previous work on O_i in $YMnO_3$,[4] this triple well potential is asymmetric as there is an additional energy gain for O_i to displace towards the O3 position, which is the trimerization center of the corner-sharing MnO_5 trigonal bipyramids.

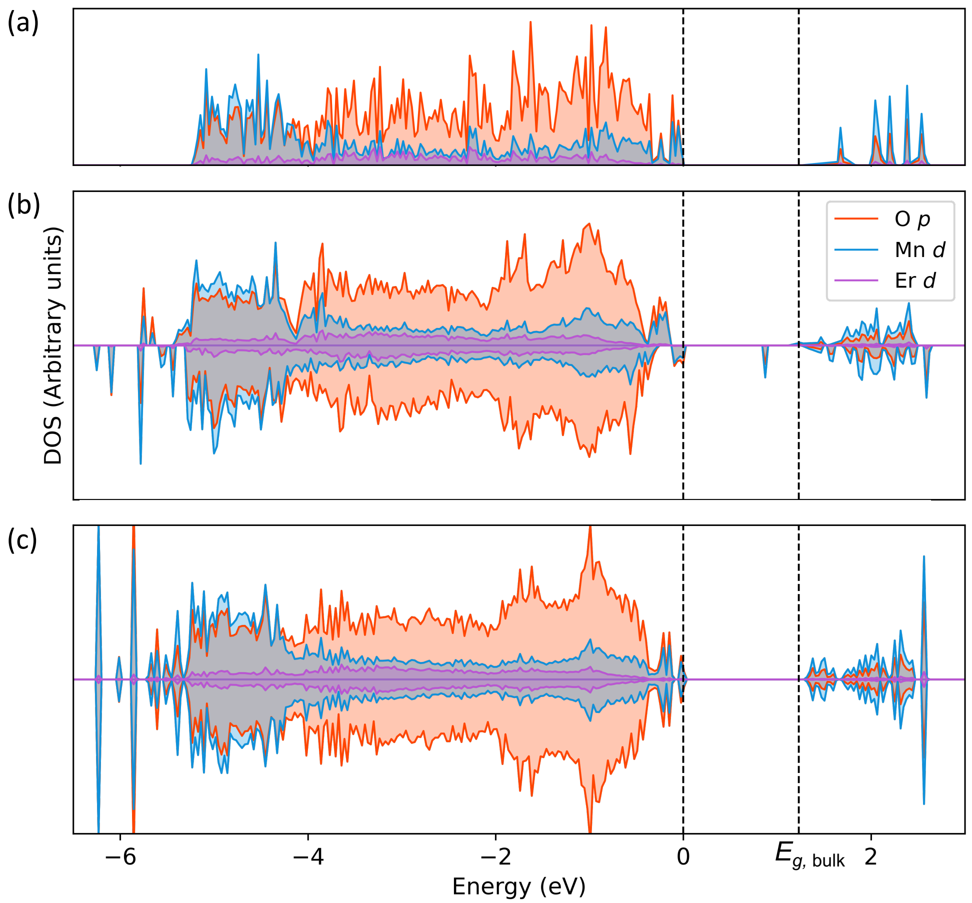

Figure S6: Density of states calculated for the 2 × 2 × 1 supercell of $ErMnO_3$ with (a) no O_i, (b) one O_i and (c) two O_i. Upper and lower plots indicate up and down spin channels, respectively, except for the case of no interstitials, as we then have perfect antiferromagnetic order. Dashed vertical lines indicate (for this plot and all following density of states) the Fermi level and bottom of the conduction band for bulk $ErMnO_3$.

The change in electronic structure from an increase in interstitial oxygen in the lattice was calculated using a $2 \times 2 \times 1$ supercell with a single O_i in a stable position, corresponding to an off-stoichiometry of $\delta = 0.04$. As displayed in Figure S6, we see the emergence of a localized and non-bonding defect state within the band gap, with the bonding states situated below the valence band. The band gap is reduced to $E_g = 0.81\,\text{eV}$. For $\delta = 0.08$, which is achieved by a single oxygen defect in each Mn−O layer, the localized nature of the defect states is diminished and they move into the bottom of the conduction band. The asymmetry in the defect states within each spin channel is also no longer present. The unfolded band structures for $\delta = 0.04$ and $\delta = 0.08$ are presented in Figure S7.

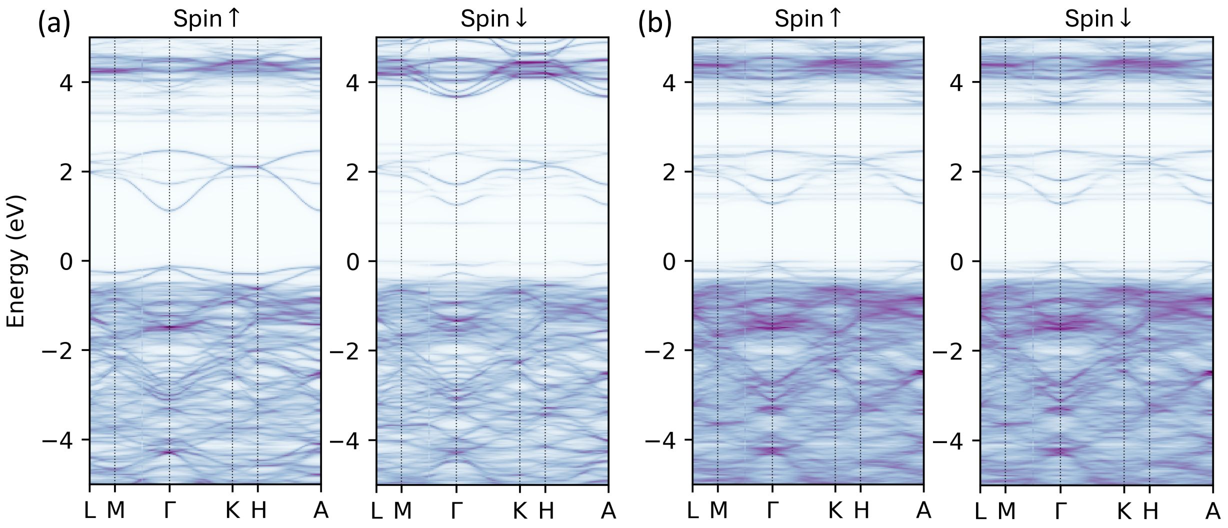

Figure S7: Unfolded band structures for the $2 \times 2 \times 1$ supercell, with (a) one O_i and (b) two O_i, separated into spin up and down channels as indicated above the plot. Defect levels appear as localized horizontal bands within and below the counduction band.

For the neutral domain wall calculations, a $1 \times 6 \times 1$ cell was used for the pristine domain wall, while a supercell of dimensions $2 \times 6 \times 1$ was used when simulating O_i adjacent to the wall to minimize defect-defect interactions. The symmetry alteration introduced by a domain wall is expected to give rise to a locally modified electronic structure as opposed to the bulk domains. However, since the polarization is parallel to a close to atomically sharp domain wall, there are no bound charges. As a result, the density of states and electronic structure

is qualitatively similar to the bulk, albeit with a slightly lower band gap of $E_g = 1.14\,\mathrm{eV}$, see Figure S8.

For simulating defects adjacent to the domain wall, all O_i were placed in stable configurations that minimized defect-defect interactions. The layer-projected density of states and unfolded band structures in the case of a single O_i adjacent to the neutral domain wall are presented in Figures S9 and S10. Both Er2-Er2 and Er1-Er1 domain walls were tested, but showed no significant differences in terms of electronic structure or formation energies, which is why only the Er2-Er2 wall is presented here. The band gap is locally lowered to $E_g = 1.03\,\mathrm{eV}$, and the defect states can be seen in both the bottom of the conduction band and valence band. For three defects adjacent to the neutral domain wall, the defect states become more localized but there is no further lowering of the band gap. We note that the frustrated collinear antiferromagnetic order introduces an artificial variation in the energy and the top and bottom of the valence and conduction bands, respectively, as discussed in the following subsection.

Frustrated collinear magnetism

Although $ErMnO_3$ is paramagnetic at room temperature, the antiferromagnetic ground state is used for DFT calculations to reduce the computational complexity. Furthermore, the non-collinear frustrated antiferromagnetism is approximated by a frustrated collinear antiferromagnetic structure (F-AFM) as this is well known to reproduce the main electronic structure features of rare earth hexagonal manganites.[5] However when introducing O_i into the crystal structure, some additional considerations need to be made when evaluating the results.

Firstly, the F-AFM order introduces an additional asymmetry to the triple well potential that the O_i experiences in bulk, as for only 1/3 of possible O_i sites the adjacent Mn^{4+} atoms have equal spin, the majority spin of that respective Mn−O layer. Placing O_i in one of the latter 2/3 positions yields an extra energy gain coming from the electronic repulsion associated with the two Mn^{4+} atoms having opposite spins. This will also result in O_i distorting such that the three Mn−O bonds are all of different lengths. Thus, for the bulk calculations, defects were placed such that the magnetic order aligned with the most stable configuration possible.

In addition to the inequality between interstitial sites stemming from the synthetic F-AFM order, the symmetry-breaking of the domain wall leads to an added artifact in terms of the position of the defect levels and ground state energy. This is because as the crystal structure switches from one domain to the other, while supporting a continuous magnetic order, each potential interstitial site experiences a different local geometry and magnetic surroundings. The relaxed O_i is thus always in a distorted structure relative to the most stable bulk position. In the case of one and three O_i adjacent to the domain wall, all configurations that were inequivalent in terms of structural and magnetic order were evaluated. The number of possible configurations for three defects are limited by restrictions on the minimum defect-defect distance. A final configuration was chosen, displaying a representative average in terms of the defect energy, as well as the location of the defect levels and thus the band gap. The variation in terms of ground state energy increases with the number of defects,

being $\Delta E_{\mathrm{O_i}} = 0.09\,\mathrm{eV}$ for one $\mathrm{O_i}$ and $\Delta E_{3\mathrm{O_i}} = 0.11\,\mathrm{eV}$ for three $\mathrm{O_i}$. An example of the variation within the position of the defect levels for three defects is presented in Figure S11. This fluctuation in the band gap results from the change in stability between the different configurations due to the domain wall naturally removing the true trimerization points, but also from the artificial magnetic order.

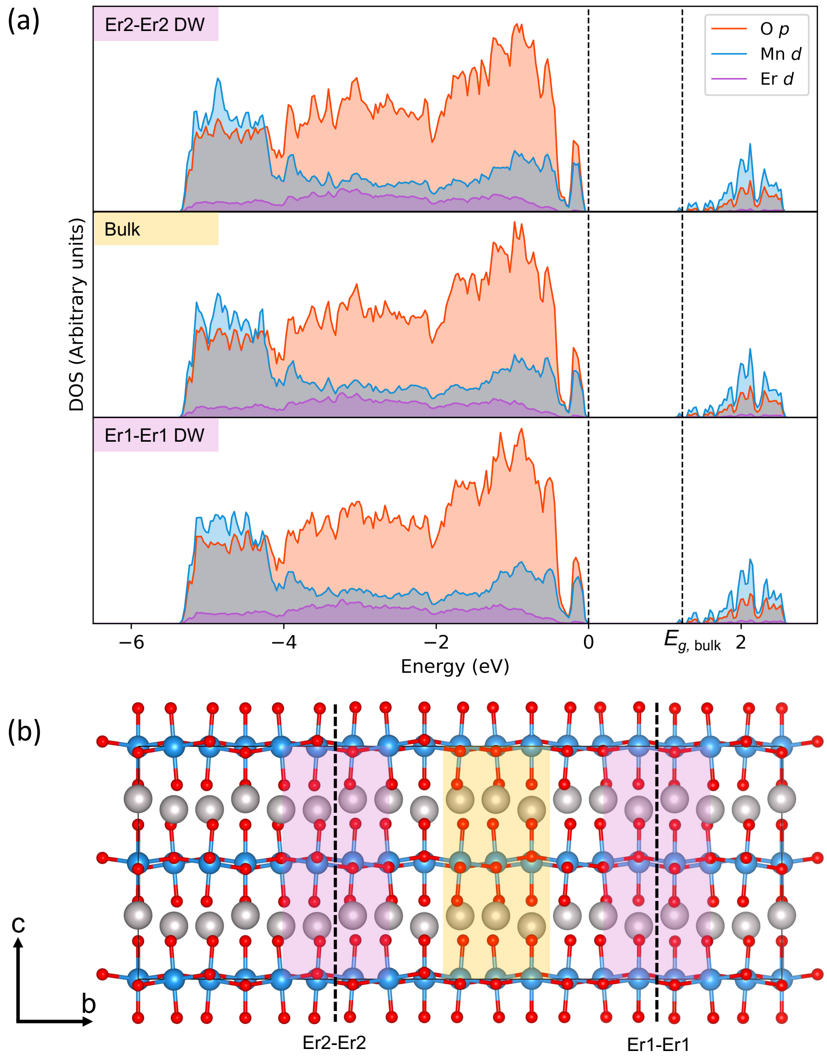

Figure S8: (a) Density of states calculated for a $1 \times 6 \times 1$ supercell with Er2-Er2 and Er1-Er1 terminated neutral domain walls. (b) Relaxed geometry of the supercell as seen from the (100) plane, with the position of each domain wall marked by vertical dashed lines. The three plots show the layer-resolved density of states for a unit cell centered at the position of each domain wall, as well as in between each wall (in domain), assigned as bulk. The position of each layer and domain wall type is indicated by the corresponding color displayed in the structure below.

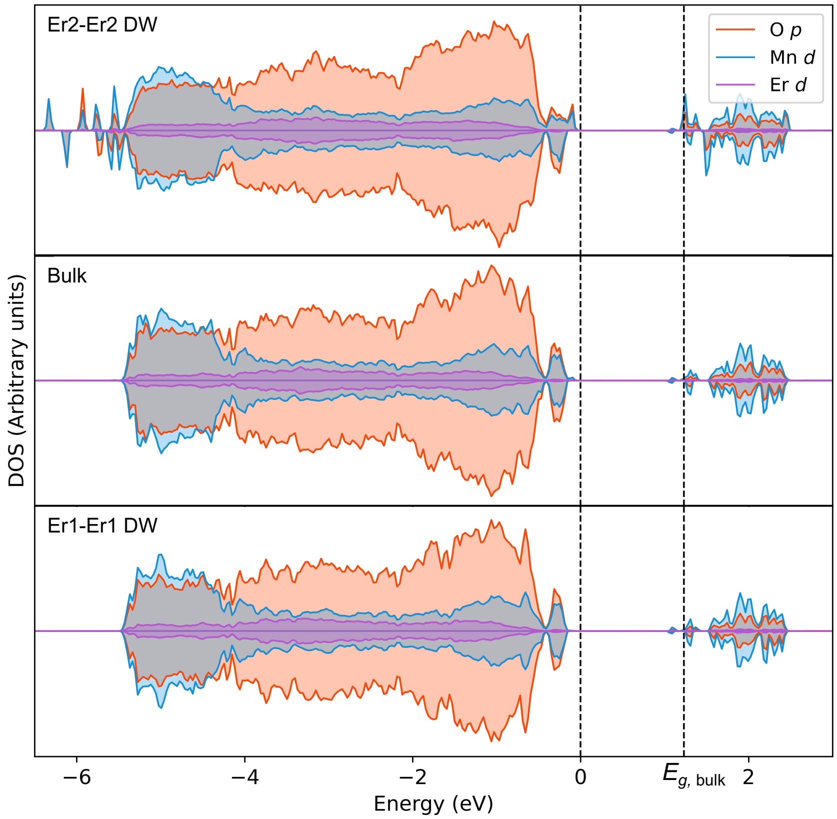

Figure S9: Layer-projected density of states calculated for a $2 \times 6 \times 1$ supercell, with a single O_i positioned adjacent to the Er2-Er2 terminated domain wall. The positions of the layers follow that of Figure S8. The defect levels attributed to the interstitials can be seen below the valence band and in the lower parts of the conduction band, in the Er2-Er2 domain wall layer.

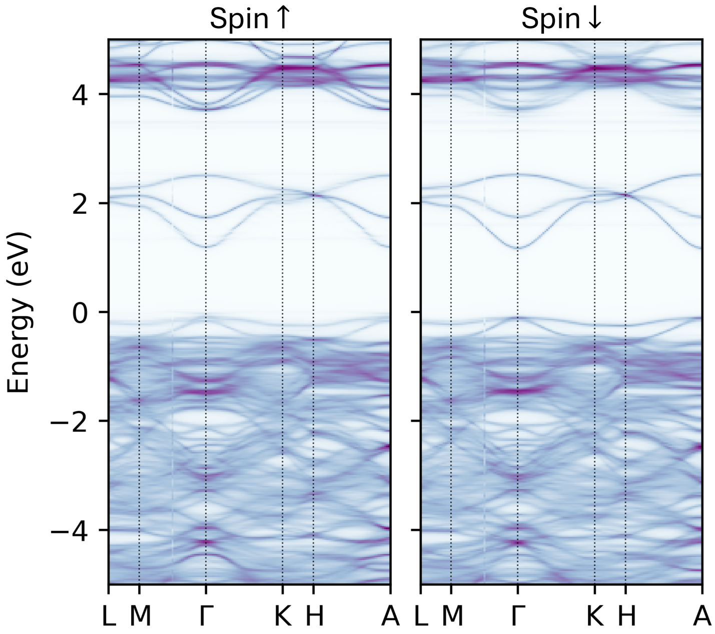

Figure S10: Unfolded band structure for spin up and down channels for the entire $2 \times 6 \times 1$ supercell, with a single oxygen interstitial adjacent to the Er2-Er2 domain wall. The localized defect states can be seen as horizontal lines within the conduction band. The low visibility of the lines results from the size of the supercell.

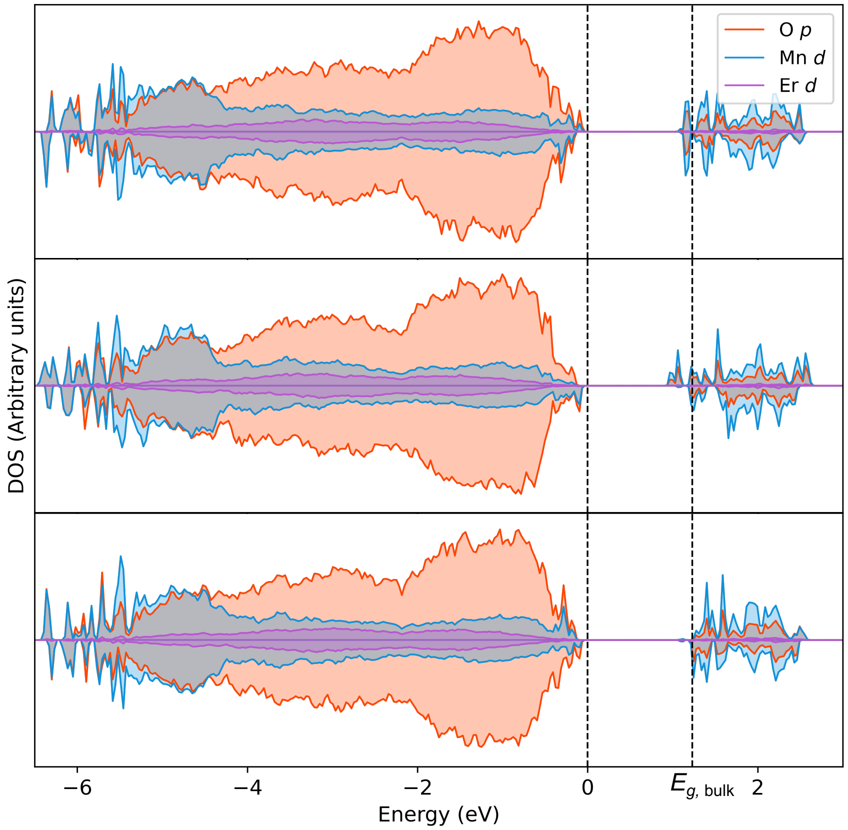

Figure S11: Density of states calculated for a $2 \times 6 \times 1$ supercell with three O_i, projected onto the unit cell centered at the Er2-Er2 domain wall. Each plot shows a potential defect configuration, along with the variation in the location of the defect levels.

References

(1) Remsen, S.; Dabrowski, B. Synthesis and Oxygen Storage Capacities of Hexagonal $Dy_{1\text{-}x}Y_xMnO_{3+\delta}$. *Chemistry of Materials* **2011**, *23*, 3818–3827.

(2) Šafránková, M.; Fousek, J.; KiŽaev, S. A. Domains in ferroelectric $YMnO_3$. *Czechoslovak Journal of Physics B* **1967**, *17*, 559–560.

(3) Wang, X.; Huang, F.-T.; Hu, R.; Fan, F.; Cheong, S.-W. Self-poling with oxygen off-stoichiometry in ferroelectric hexagonal manganites. *APL Materials* **2015**, *3*, 041505.

(4) Skjærvø, S. H.; Wefring, E. T.; Nesdal, S. K.; Gaukås, N. H.; Olsen, G. H.; Glaum, J.; Tybell, T.; Selbach, S. M. Interstitial oxygen as a source of p-type conductivity in hexagonal manganites. *Nature Communications* **2016**, *7*, 13745.

(5) Medvedeva, J. E.; Anisimov, V. I.; Korotin, M. A.; Mryasov, O. N.; Freeman, A. J. The effect of Coulomb correlation and magnetic ordering on the electronic structure of two hexagonal phases of ferroelectromagnetic $YMnO_3$. *Journal of Physics: Condensed Matter* **2000**, *12*, 4947.
